# Faricimab in neovascular age-related macular degeneration: aqueous humor cytokine profiling and biomarker correlations

**DOI:** 10.3389/fmed.2026.1927909

**Published:** 2026-08-21

**Authors:** Zhongqi Wan, Xinxia Chen, Fei Gao, Fei Shi, Rushun Dai, Qing Peng, Qingquan Wei

**Affiliations:** 1 Department of Ophthalmology, Shanghai Tenth People’s Hospital, School of Medicine, Tongji University, Shanghai, China; 2 Department of Ophthalmology, Shanghai Heping Eye Hospital, Shanghai, China; 3 MIPAV Lab, School of Electronic and Information Engineering, Soochow University, Suzhou, China; 4 Department of Clinical Laboratory, Shanghai Tenth Hospital, Tongji University School of Medicine, Shanghai, China; 5 Department of Ophthalmology, Tongren Hospital, Shanghai Jiao Tong University School of Medicine, Shanghai, China

**Keywords:** Angiopoietin-2, biomarkers, inflammatory factors, neovascular age-related macular degeneration, vascular endothelial growth factor

## Abstract

**Purpose:**

To investigate aqueous humor cytokine profile changes in neovascular age-related macular degeneration (nAMD) patients before and after intravitreal faricimab injection, and their correlation with biomarkers.

**Methods:**

Twenty-three nAMD patients and 10 cataract controls were enrolled. Aqueous humor was collected from nAMD patients at baseline and 12 weeks post-faricimab injection, and from controls during phacoemulsification. Twelve cytokines were quantified via multiplex bead-based immunoassay. OCTA measured central macular thickness (CMT) and retinal vessel density in nAMD eyes.

**Results:**

Baseline vascular endothelial growth factor (VEGF), Angiopoietin-2 (Ang-2) and HGF levels were higher in nAMD patients than controls. Post-treatment, VEGF and Ang-2 decreased below control levels, while hepatocyte growth factor (HGF) and interferon gamma protein 10 (IP-10) increased significantly. Reductions in Ang-2/VEGF correlated positively with decreased CMT and whole retinal thickness, with concurrent improvements in best-corrected visual acuity. Elevated IP-10 showed a correlative association with reduced superficial foveal vessel density.

**Conclusion:**

Faricimab dual-blockades VEGF/Ang-2, reducing their aqueous levels, improving CMT/whole retinal thickness and visual acuity. However, post-treatment HGF/IP-10 upregulation indicates compensatory inflammatory-angiogenic remodeling. IP-10 may serve as a potential correlative biomarker linked to subclinical capillary rarefaction. Future studies should explore combined anti-IP-10 therapy or extended dosing intervals to preserve foveal vasculature while maintaining anti-leakage efficacy.

## Introduction

Neovascular age-related macular degeneration (nAMD) is the leading cause of irreversible central vision loss in individuals aged ≥60 years globally (1). Although intravitreal inhibition of vascular endothelial growth factor (VEGF) has significantly improved patient outcomes, a considerable proportion of patients still require frequent injections or develop tolerance, indicating that other angiogenic and inflammatory pathways beyond VEGF continue to drive choroidal neovascularization (CNV) and vascular hyperpermeability (2, 3).

Angiopoietin-2 (Ang-2) is significantly upregulated in the vitreous fluid of patients with retinal vascular diseases, acting together with VEGF-A as a synergistically regulated pathogenic mediators (4). The simultaneous inhibition of VEGF-A and Ang-2 yields superior therapeutic outcomes compared to anti-VEGF-A monotherapy (4, 5). In spontaneous CNV models, dual inhibition of VEGF-A and Ang-2 significantly reduces the number of vascular lesions, vascular permeability, retinal edema, and neuronal loss (5–7).

Faricimab is a novel bispecific antibody targeting both VEGF and Ang-2, offering potential therapeutic advantages over monotherapy by addressing multiple pathways involved in nAMD (8). Previous studies have demonstrated the efficacy and safety of faricimab in treating nAMD; however, detailed changes in the aqueous humor cytokine profile and their correlations with clinical biomarkers remain unclear (9).

The aqueous humor is in direct contact with ocular tissues and can reflect local inflammatory and angiogenic status (10). Optical coherence tomography angiography (OCTA), as a non-invasive imaging modality, provides detailed information on retinal and choroidal vasculature, enabling the assessment of CNV activity and retinal structural changes (11, 12). By combining aqueous humor cytokine profiling with OCTA parameters, we can elucidate the pathophysiological changes in nAMD and the therapeutic effects of faricimab.

Therefore, this study aimed to comprehensively analyze changes in the aqueous humor cytokine profile in patients with nAMD before and after intravitreal faricimab injection and to explore their correlations with clinical biomarkers, including OCTA parameters. This research provides valuable information for optimizing nAMD treatment strategies and further understanding the complex pathogenesis of this blinding disease.

## Methods

### Study design and participants

This prospective observational study enrolled 23 patients with nAMD and 10 age-matched cataract controls who visited the Department of Ophthalmology, Shanghai Tenth People’s Hospital between March 2024 and March 2025. The study protocol was approved by the Institutional Review Board of Shanghai Tenth People’s Hospital, Tongji University School of Medicine (ChiCTR2100051795) and adhered to the tenets of the Declaration of Helsinki. Written informed consent was obtained from all the participants.

Post-hoc power analysis was performed to evaluate the statistical robustness of the current sample size (23 nAMD patients and 10 cataract controls). The analysis revealed that the small control group yielded limited statistical power to establish highly stable aqueous cytokine baseline references, which constitutes an inherent limitation of this single-center observational study. Patient and control recruitment was restricted to a fixed 12-month enrollment window (March 2024–March 2025). After the recruitment period closed, no additional eligible age-matched cataract patients meeting all inclusion/exclusion criteria could be recruited for supplementary aqueous humor sampling, so further expansion of the control cohort was not feasible.

### Inclusion and exclusion criteria

The inclusion criteria for the nAMD group were as follows: (1) age of 50–75 years; (2) presence of active CNV located subfoveally or juxtafoveally in the study eye; (3) best-corrected visual acuity (BCVA) of the study eye between 19 and 73 ETDRS letters (equivalent to Snellen visual acuity of 20/400–20/40); (4) no intravitreal injection of anti-VEGF drugs, photodynamic therapy, or retinal photocoagulation in the study eye within the past 3 months; and (5) no systemic or local use of corticosteroids or dexamethasone within the past 3 months. All enrolled participants were treatment-naive nAMD patients without any history of prior intravitreal anti-angiogenic injection, photodynamic therapy or retinal laser photocoagulation before study entry; no patients switched from other anti-VEGF agents were recruited in this cohort. The exclusion criteria were: (1) ≥ 50% of the lesion area in the study eye covered by scar or fibrotic tissue, or the presence of subfoveal scar, fibrosis, or atrophy; (2) active ocular infection in either the study eye or the fellow eye; (3) CNV caused by non-AMD diseases (e.g., pathologic myopia), or the presence of any non-nAMD ocular condition (e.g., diabetic retinopathy, central retinal vein occlusion, and glaucoma) that could affect macular examination or central vision, either currently or previously; (4) active infection requiring oral, intramuscular, or intravenous medication; (5) active disseminated intravascular coagulation or significant bleeding tendency within the past 3 months; (6) systemic autoimmune disease; and (7) any uncontrolled clinical condition (such as severe psychiatric, neurological, cardiovascular, respiratory diseases, or malignancy).

The exclusion criteria for the cataract control group were as follows: (1) coexistence of other ocular diseases and history of any vitreous hemorrhage within the past 3 months; (2) active infection requiring oral, intramuscular, or intravenous medication; (3) active disseminated intravascular coagulation or significant bleeding tendency within the past 3 months; (4) systemic autoimmune disease; and (5) any uncontrolled clinical condition (such as severe psychiatric, neurological, cardiovascular, or respiratory diseases, or malignancy).

### Intervention and sample collection

All intravitreal injections were administered by a single experienced surgeon, using the 4 + PRN faricimab regimen. Topical 0.5% levofloxacin q.i.d. was started 3 days injection. After topical 0.5% proparacaine (three drops), the periocular area and lashes were disinfected with 0.5% povidone-iodine twice; the conjunctival sac was irrigated with 0.05% povidone-iodine for 60 s, followed by 2% lidocaine. A 30 G needle was inserted 4 mm posterior to the limbus, and 0.05 mL faricimab (Vabysmo®, Roche/Regeneron) was injected. Digital pressure was applied for 1–3 min, and TobraDex ointment and an eye patch were placed postoperatively.

Aqueous humor (≈100 μL) was obtained by limbal paracentesis with a 30 G needle immediately before the first and third injections. An equivalent volume was collected from the cataract controls at the beginning of phacoemulsification. Samples were transferred to pre-labeled 1.5 mL Eppendorf tubes and stored at −80 °C until analysis. Serial sampling at 4 weeks and 8 weeks was not performed due to two critical clinical constraints in this single-center cohort. Repeated limbal paracentesis for serial aqueous collection carries cumulative invasive risks including conjunctival irritation, corneal epithelial injury and minor intraocular irritation. Meanwhile, many elderly nAMD participants could not sustain monthly hospital visits across the full 12-month recruitment cycle, resulting in poor follow-up compliance for intermediate sampling. Together, these barriers prevented us from implementing 4-week and 8-week specimen collection and restrict our ability to capture the complete continuous kinetic trajectory of intraocular cytokines throughout treatment.

### Cytokine analysis

Twelve cytokines were quantified using the Bio-Plex™ 200 suspension array system (Bio-Rad) and the Luminex® Discovery Assay Human Premixed Multi-analyte Kit: Ang-2, VEGF-A, hepatocyte growth factor (HGF), platelet-derived growth factor-AA (PDGF-AA), interferon gamma protein 10 (IP-10), basic fibroblast growth factor (FGF-basic), heparin-binding epidermal growth factor-like growth factor (HB-EGF), tumor necrosis factor alpha (TNF-*α*), interleukin (IL)-1β, IL-6, IL-8, and placental growth factor (PlGF). All assays were performed in duplicate according to the manufacturer’s instructions, and the median fluorescence intensities were converted to pg/mL using standard curves.

The 12 analytes in this multiplex panel were selected for clear biological and technical reasons. VEGF-A and Ang-2 are the direct molecular targets of faricimab. The remaining growth factors (HGF, PDGF-AA, FGF-basic, HB-EGF, PlGF) and inflammatory chemokines/cytokines (IP-10, TNF-*α*, IL-1β, IL-6, IL-8) are well-established key mediators of choroidal neovascularization and intraocular inflammation in nAMD according to published literature. Besides biological relevance, this pre-mixed detection kit is widely validated for ocular fluid research; given the limited volume of aqueous humor (~100 μL per sample), we adopted this mature 12-plex panel to avoid overconsumption of limited biological samples and ensure comparability with prior biomarker studies.

### Ophthalmic examinations

Patients with nAMD underwent a comprehensive ocular assessment (BCVA, slit-lamp, tonometry, fundus photography, and OCTA) at baseline and immediately before the fourth injection. BCVA was recorded using ETDRS charts and converted to LogMAR. OCTA (Optovue RTVue-XR, USA) 6 × 6 mm macular scans were analyzed for central macular thickness (CMT), whole retinal thickness, superficial retinal vessel density (SRVD), deep retinal vessel density (DRVD), superficial foveal vessel density, deep foveal vessel density (DFVD), and foveal avascular zone (FAZ) using an experienced masked grader.

### Study outcomes

Primary outcome measures included: (1) comparison of aqueous cytokine levels between the nAMD and cataract groups; (2) longitudinal changes in cytokines after faricimab treatment; (3) changes in BCVA, CMT, whole retinal thickness, SRVD, DRVD, superficial foveal vessel density, DFVD, and FAZ; (4) correlations between cytokines (especially Ang-2) and OCTA parameters; and (5) inter-cytokine relationships within groups.

### Statistical analysis

SPSS 23.0 was used. Normally distributed continuous data are presented as means ± standard deviations and skewed data as medians (P25, P75). Between-group comparisons: independent t-test or Mann–Whitney U test. For pre- vs. post-treatment comparisons, paired t-test or Wilcoxon signed-rank test was used. Correlations between cytokines and OCTA parameters were evaluated using Spearman’s rho; two-tailed *p* < 0.05 was considered significant. Longitudinal cytokine changes and IP-10 vs. SRVD correlations were visualized using box and scatter plots generated in R 4.2.0.

## Results

### Baseline characteristics

This study included 23 patients (23 eyes) in the nAMD group, comprising 12 men and 11 women, with a mean age of 73.39 ± 3.46 years. This group comprised 11 right and 12 left eyes. Additionally, the cataract control group included 10 patients (10 eyes), comprising 5 men and 5 women, with a mean age of 75.26 ± 2.88 years. The control group consisted of six right and four left eyes. No significant differences in age, sex, or eye distribution were observed between the nAMD and cataract control groups. No significant differences were observed in the prevalence of diabetes or hypertension between the nAMD and control groups (Table 1). Of note, these intergroup cytokine comparisons should be interpreted with caution, as the limited sample size of the cataract control group may lead to unstable baseline cytokine concentrations and potential statistical fluctuation.

**Table 1:** Demographic characteristics of the patients in the nAMD and control groups.

Characteristics	nAMD	Cataract	*p* value
Participants (*n*)	23	10	–
Age (years)	73.39 ± 3.46	75.26 ± 2.88	>0.05
Sex (M/F)	12/11	5/5	>0.05
Eye (right/left)	11/12	6/4	>0.05
Hypertension (*n*, %)	3 (13.0%)	1 (10.0%)	>0.05
Diabetes (*n*, %)	2 (8.7%)	1 (10.0%)	>0.05

M/F = male/female. Data are presented as means ± standard deviations of each group; *p* < 0.05 was considered statistically significant.

### Changes in the aqueous cytokine profile

Before the first injection, eyes with nAMD exhibited significantly higher aqueous concentrations of VEGF, HGF, and Ang-2 than cataract controls. Baseline levels of PDGF-AA, IP-10, FGF-basic, HB-EGF, TNF-*α*, IL-1β, IL-6, IL-8, and PlGF did not significantly differ between the two groups. Following intravitreal faricimab administration, VEGF and Ang-2 levels in the nAMD group were lower than those in the control eyes, whereas HGF levels increased significantly above the control values (*p* < 0.05). PDGF-AA, IP-10, FGF-basic, HB-EGF, TNF-*α*, IL-1β, IL-6, IL-8, and PlGF remained comparable between groups after treatment. Longitudinal analysis within the nAMD cohort confirmed that faricimab markedly reshaped the cytokine milieu; VEGF and Ang-2 decreased substantially, whereas IP-10 increased significantly, relative to baseline. No significant changes were observed for PDGF-AA, FGF-basic, HB-EGF, TNF-*α*, IL-1β, IL-6, IL-8, or PlGF (Table 2).

**Table 2:** Cytokine concentration in aqueous humor before and after treatment in the nAMD group and cataract group (pg/ml).

Variable	Cataract	Pre-nAMD	Post-nAMD	*P*_1_	*P*_2_	*P*_3_
Ang-2	44.21 ± 18.20	63.13 ± 18.82	19.71 ± 9.34	0.014*	<0.001***	<0.001***
FGF basic	6.14 ± 2.96	8.71 ± 7.37	9.42 ± 6.79	0.311	0.165	0.740
HGF	188.52 ± 67.91	262.52 ± 97.73	342.38 ± 192.16	0.043*	0.023*	0.089
IL-6	22.74 ± 20.53	21.77 ± 51.04	38.64 ± 57.59	0.955	0.416	0.309
PDGF-AA	21,13 ± 6.97	23.62 ± 6.78	23.31 ± 6.61	0.358	0.412	0.878
TNF-α	0.65 ± 0.01	0.65 ± 0.27	0.57 ± 0.33	0.972	0.500	0.406
IP-10	37.02 ± 24.86	37.76 ± 25.44	71.72 ± 68.05	0.940	0.139	0.034*
HBEGF	3.84 ± 1.05	2.63 ± 1.15	2.78 ± 1.44	0.083	0.186	0.780
IL-1β	2.73 ± 0.38	2.74 ± 0.65	2.58 ± 0.37	0.980	0.314	0.335
IL8	8.77 ± 5.56	7.67 ± 6.14	11.67 ± 7.47	0.640	0.293	0.058
PlGF	0.71 ± 0.26	0.71 ± 0.34	0.89 ± 0.42	0.953	0.232	0.111
VEGF	62.95 ± 38.25	122.77 ± 67.86	8.06 ± 7.39	0.017*	<0.001***	<0.001***

P_1_ (pre-nAMD vs. cataract); P_2_ (post-nAMD vs. cataract); P_3_ (post-nAMD vs. pre-nAMD). *indicates *p* < 0.05, ** indicates *p* < 0.01, *** indicates *p* < 0.001.

### Analysis of cytokine correlations

Before treatment, the nAMD cohort exhibited a tightly interlinked pro-angiogenic and pro-inflammatory network. Ang-2 correlated strongly with VEGF (r = 0.78), whereas HGF was closely associated with IL-6 (r = 0.745), IP-10 (r = 0.85), HB-EGF (r = 0.829), and IL-8 (r = 0.862). IL-6 in turn showed robust positive relationships with IP-10 (r = 0.781), HB-EGF (r = 0.829), and IL-8 (r = 0.813). IP-10 was highly correlated with both HB-EGF (r = 0.808) and IL-8 (r = 0.761). HB-EGF was significantly linked to IL-8 (r = 0.603) (Table 3).

**Table 3:** Correlation among cytokines before treatment in the nAMD group.

Variable	Ang-2	FGF basic	HGF	IL-6	PDGF-AA	TNF-α	IP-10	HB-EGF	IL-1β	IL-8	PIGF	VEGF
Ang-2		−0.010 (0.946)	0.187 (0.393)	−0.028 (0.899)	−0.138 (0.529)	−0.095 (0.666)	0.063 (0.774)	0.297 (0.168)	0.282 (0.193)	0.110 (0.618)	−0.139 (0.526)	0.697 (0.000***)
FGF basic			−0.332 (0.121)	−0.278 (0.199)	0.007 (0.973)	0.146 (0.507)	−0.184 (0.401)	−0.312 (0.147)	−0.287 (0.184)	−0.232 (0.286)	0.077 (0.727)	0.111 (0.614)
HGF				0.730 (0.000***)	−0.003 (0.988)	−0.053 (0.809)	0.727 (0.000***)	0.725 (0.000***)	0.206 (0.347)	0.818 (0.000***)	0.224 (0.305)	0.053 (0.809)
IL-6					0.074 (0.736)	−0.014 (0.949)	0.801 (0.000***)	0.525 (0.010*)	0.094 (0.670)	0.746 (0.000***)	0.409 (0.053)	−0.051 (0.816)
PDGF- AA						0.118 (0.591)	0.301 (0.162)	0.118 (0.591)	0.003 (0.989)	−0.040 (0.856)	−0.319 (0.137)	−0.295 (0.172)
TNF-α							−0.272 (0.210)	0.021 (0.924)	0.291 (0.178)	−0.023 (0.917)	−0.024 (0.912)	−0.333 (0.121)
IP-10								0.562 (0.005**)	−0.031 (0.889)	0.722 (0.000***)	0.197 (0.368)	0.093 (0.673)
HB-EGF									0.256 (0.238)	0.648 (0.001**)	0.005 (0.983)	0.151 (0.490)
IL-1β										0.041 (0.851)	−0.211 (0.333)	−0.029 (0.894)
IL-8											0.272 (0.209)	0.042 (0.849)
PIGF												0.065 (0.770)

Values in each cell represent Spearman’s correlation coefficient (r), with corresponding *p*-values shown in parentheses. *indicates *p* < 0.05, ** indicates *p* < 0.01, *** indicates *p* < 0.001.

Following faricimab injection, the correlation landscape was markedly reconfigured. Ang-2 expression was positively correlated with HGF (r = 0.569), IP-10, PlGF (r = 0.560), and VEGF (r = 0.675). HGF maintained strong correlations with IL-6 (r = 0.736), TNF-*α* (r = 0.599), IP-10 (r = 0.831), HB-EGF (r = 0.591), and IL-8 (r = 0.560). IL-6 continued to correlate closely with IP-10 (r = 0.862), HB-EGF (r = 0.590), and IL-8 (r = 0.809), whereas TNF-α was positively related to IP-10 (r = 0.619). Additionally, IP-10 was strongly associated with HB-EGF (r = 0.618) and IL-8 (r = 0.793), and HB-EGF was significantly correlated with IL-8 (r = 0.734) (Table 4).

**Table 4:** Correlation among cytokines after treatment in the nAMD group.

Variable	Ang-2	FGF basic	HGF	IL-6	PDGF-AA	TNF-α	IP-10	HB-EGF	IL-1β	IL-8	PIGF	VEGF
Ang-2		0.358 (0.094)	0.595 (0.003**)	0.369 (0.083)	0.257 (0.236)	0.299 (0.165)	0.417 (0.048*)	0.293 (0.175)	−0.077 (0.727)	0.231 (0.289)	0.511 (0.013*)	0.523 (0.010*)
FGF basic			0.146 (0.505)	0.066 (0.764)	−0.070 (0.750)	0.188 (0.391)	0.259 (0.233)	−0.058 (0.792)	−0.216 (0.323)	0.160 (0.467)	0.172 (0.433)	0.108 (0.623)
HGF				0.686 (0.000***)	0.248 (0.253)	0.421 (0.045*)	0.711 (0.000***)	0.456 (0.029*)	0,071 (0.747)	0.454 (0.029*)	0.377 (0.076)	0.272 (0.209)
IL-6					0.318 (0.139)	0.362 (0.090)	0.816 (0.000***)	0.550 (0.007**)	0.274 (0.206)	0.678 (0.000***)	0.174 (0.429)	0.065 (0.768)
PDGF- AA						0.250 (0.251)	0.247 (0.255)	0.021 (0.926)	0.027 (0.904)	−0.142 (0.518)	0.134 (0.542)	−0.146 (0.506)
TNF-α							0.569 (0.005**)	0.186 (0.396)	0.061 (0.783)	0.380 (0.074)	0.160 (0.466)	−0.039 (0.859)
IP-10								0.563 (0.005**)	0.055 (0.802)	0.791 (0.000***)	0.215 (0.325)	0.215 (0.324)
HB-EGF									0.371 (0.082)	0.706 (0.000***)	0.156 (0.478)	0.202(0.356)
IL-1β										0.306 (0.155)	0.034 (0.877)	0.007 (0.976)
IL-8											0.287 (0.185)	0.133 (0.546)
PIGF												0.405 (0.055)

Values in each cell represent Spearman’s correlation coefficient (r), with corresponding *p*-values shown in parentheses. *indicates *p* < 0.05, ** indicates *p* < 0.01, *** indicates *p* < 0.001.

### Changes in OCTA parameters and clinical outcomes

**Figure 1:**
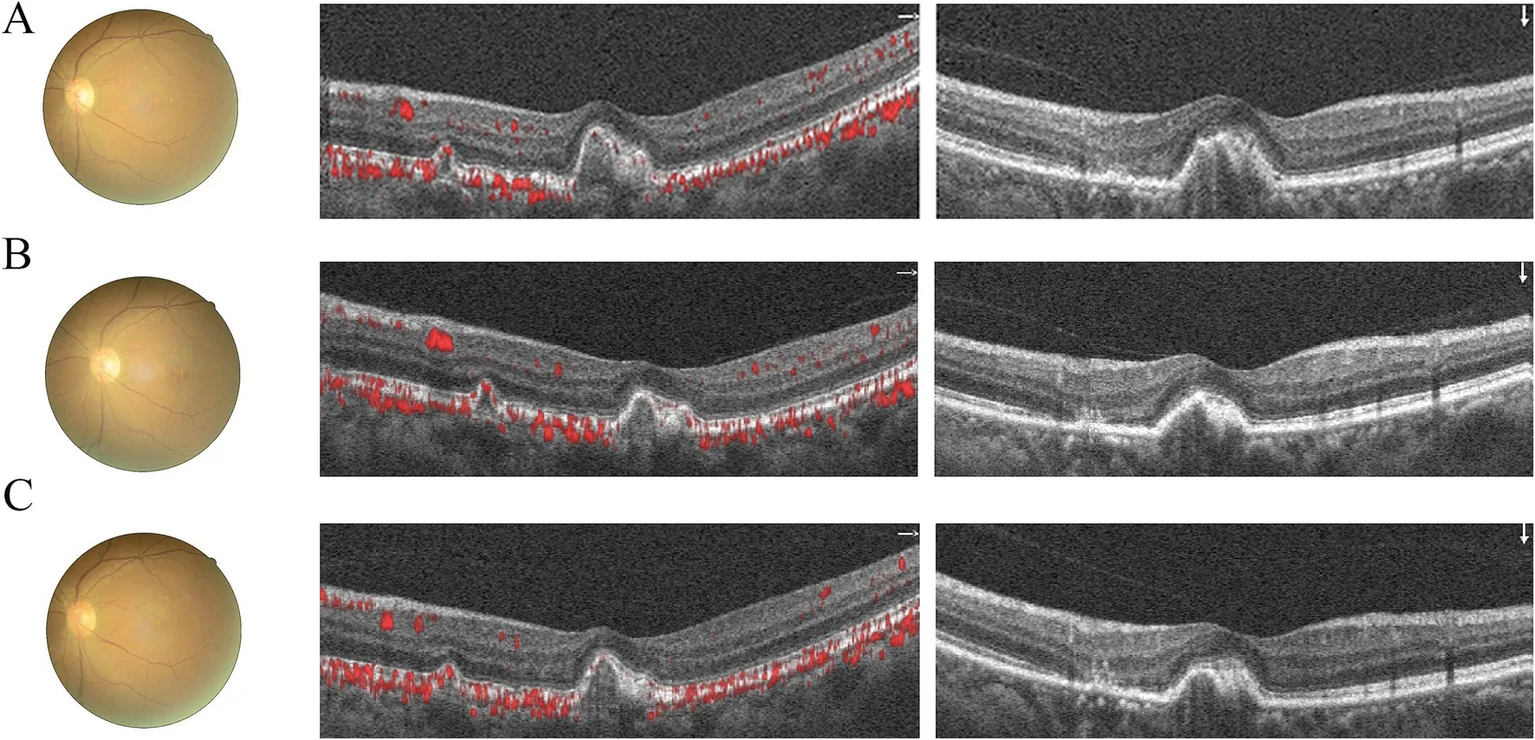
Serial fundus photographs and OCTA macular scans (6 × 6 mm standardized macular scanning protocol, built-in instrument scale bar for retinal vascular quantification) from a representative nAMD patient receiving intravitreal faricimab. **(A)** Preoperative fundus photograph and OCTA at 1 week; **(B)** Postoperative fundus photograph OCTA at 1 month; **(C)** Postoperative fundus photograph and OCTA at 3 months. The 6 × 6 mm scanning mode centered on the fovea fully covers the central macular region where choroidal neovascularization lesions frequently occur, with unified built-in scale for consistent vessel density measurement. OCTA, optical coherence tomography angiography.

OCTA revealed significant anatomic improvement after faricimab therapy, and whole retinal thickness and CMT decreased markedly. By contrast, superficial foveal vessel density was reduced relative to the baseline, whereas all other vascular density parameters remained unchanged. Additionally, BCVA showed a significant improvement compared to baseline (Table 5). Figure 1 illustrates the response of a typical patient with nAMD to intravitreal faricimab therapy, showing changes in fundus photography and OCT.

**Table 5:** Comparison of OCTA parameters before and after treatment in nAMD eyes.

Variable	Pre-nAMD	Post-nAMD	*P* value
BCVA (logMAR)	0.88 ± 0.39	0.46 ± 0.36	0.000***
SRVD	41.5 ± 8.1	40.0 ± 6.1	0.613
DRVD	42.0 ± 7.7	39.2 ± 6.3	0.322
Superficial foveal vessel density	30.2 ± 11.0	15.7 ± 7.5	0.003**
DFVD	44.6 ± 17.2	36.6 ± 17.5	0.312
FAZ	0.5 ± 0.9	0.4 ± 0.3	0.758
Whole retinal thickness (μm)	309.1 ± 43.5	273.6 ± 30.1	0.040*
CMT (μm)	459.0 ± 167.6	297.5 ± 68.2	0.011*

BCVA, best-corrected visual acuity; SRVD, superficial retinal vessel density; DRVD, deep retinal vessel density; DFVD, deep foveal vessel density; CMT, central macular thickness. *indicates *p* < 0.05, ** indicates *p* < 0.01, *** indicates *p* < 0.001.

### Correlations between cytokines and OCTA parameters

**Figure 2:**
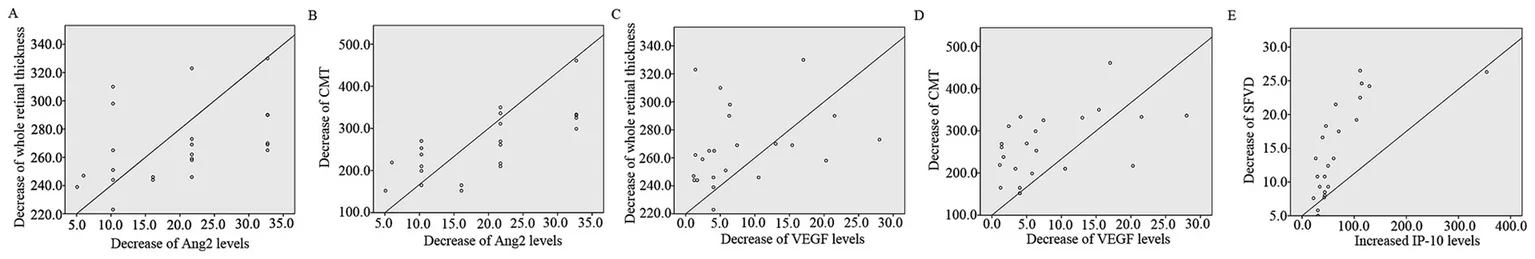
Correlations between cytokines and OCTA parameters at 3 months after intravitreal injection of Faricimab. **(A)** Significant positive correlation between decreased Ang-2 level and whole retinal thickness. **(B)** Significant positive correlation between decreased Ang-2 level and decreased CMT. **(C)** Significant positive correlation between decreased VEGF level and whole retinal thickness. **(D)** Significant positive correlation between decreased VEGF level and decreased CMT. **(E)** Significant positive correlation between increased IP-10 level and decreased superficial foveal vessel density. OCTA, optical coherence tomography angiography; CMT, central macular thickness; VEGF, vascular endothelial growth factor.

Correlation analysis between cytokine levels and OCTA parameters revealed that a decrease in Ang-2 levels was significantly and positively correlated with a reduction in whole retinal thickne0.729ss (r = 0.521, *p* = 0.011) and CMT (r = 0.729, *p* < 0.001). Similarly, the decrease in VEGF levels showed a significant positive correlation with the reduction in whole retinal thickness (r = 0.443, *p* = 0.034) and CMT (r = 0.523, *p* = 0.010). In addition, we observed a significant correlative association between elevated IP-10 levels and reduced superficial foveal vessel density (r = 0.808, *p* < 0.001, Figure 2). Statistical correlation data between all cytokines and OCT/OCTA parameters are shown in Supplementary Table 1.

## Discussion

By analyzing the dynamic changes in aqueous humor cytokine profiles before and after intravitreal faricimab injection in patients with nAMD and integrating OCTA imaging biomarkers, this study elucidates the mechanisms of action and clinical efficacy of this novel bispecific antibody. Our core finding was that faricimab effectively achieved its design goal by simultaneously inhibiting the VEGF and Ang-2 pathways, leading to significant anatomical and visual improvements. However, the treatment also triggered complex cytokine network remodeling, particularly a compensatory increase in HGF and significant upregulation of IP-10, revealing additional layers of disease pathophysiology and treatment responses.

First, our study directly corroborates the pharmacological action of faricimab. Before treatment, the aqueous humor levels of VEGF and Ang-2 in eyes with nAMD were significantly higher than those in the control group, consistent with their central role in driving pathological angiogenesis and vascular leakage, though this intergroup difference requires further verification in expanded control cohorts (13, 14). After treatment, the concentrations of both rapidly decreased to levels even below those of the cataract control group, aligning with its potent dual-blocking capability (15). More importantly, the extent of reduction in Ang-2 and VEGF levels showed significant positive correlations with the decrease in CMT and whole retinal thickness, respectively. This finding holds crucial clinical significance as it links, for the first time at the aqueous molecular level, the target-inhibition effect of faricimab with the key anatomical improvements (edema resolution) observed using OCTA, providing direct mechanistic evidence for its superior anti-leakage efficacy.

Second, this study revealed the compensatory and adaptive cytokine responses induced by the treatment. Despite the effective inhibition of VEGF/Ang-2, HGF levels increased further after treatment. HGF is a potent pro-angiogenic factor, and its upregulation may represent an “escape” mechanism inherent to the disease or triggered by therapy (16, 17). Thus, the HGF pathway could be a potential supplementary therapeutic target in patients with suboptimal responses to anti-VEGF/Ang-2 monotherapy. Concurrently, we observed a significant posttreatment increase in IP-10 levels. IP-10 is generally considered to have antiangiogenic and antifibrotic properties, and its upregulation may be an endogenous feedback mechanism against excessive angiogenesis (18, 19). However, we only observed a significant cross-sectional correlative link between elevated IP-10 levels and reduced superficial foveal vessel density, which merely offers preliminary observational evidence rather than definitive causal proof. It remains speculative that excessive IP-10 upregulation could be linked to treatment-associated subclinical foveal capillary rarefaction. We hypothesize that IP-10 could act as a sensitive correlative biomarker to monitor possible microvascular adverse changes including capillary non-perfusion, yet direct mediation of capillary loss by IP-10 cannot be confirmed based solely on the present observational data. Notably, the concurrent changes of IP-10 and superficial foveal vessel density captured in this cohort only deliver preliminary associative evidence. At this stage, we cannot verify that IP-10 directly mediates capillary loss; this proposed mechanistic pathway remains a tentative hypothesis and demands targeted interventional research, mechanistic cell or animal experiments, and larger prospective cohorts for definitive validation.

To partially offset the limitation that only baseline and 12-week aqueous samples were collected in our cohort, we compared our longitudinal cytokine alterations with published serial-sampling studies of anti-VEGF and faricimab therapy that implemented 4-week and 8-week intermediate timepoints (20). A systematic meta-analysis of aqueous cytokine research in nAMD demonstrated that HGF and IP-10 only show transient mild compensatory elevation at weeks 4–8 after single anti-VEGF injection, with partial attenuation by week 12, representing short-term acute feedback signals (21). In contrast, our 12-week data revealed persistent upregulation of both HGF and IP-10 rather than spontaneous regression, a consistent phenomenon also observed in subgroup analyses of patients switched to faricimab with sampling at 7–12 weeks (22). This discrepancy implies that simultaneous dual inhibition of VEGF and Ang-2 triggers longer-lasting inflammatory and angiogenic remodeling than single VEGF blockade. Nevertheless, without intermediate 4- and 8-week samples within our own cohort, we cannot fully trace the complete kinetic curve of these compensatory factors, nor definitively distinguish short-lived transient spikes from sustained long-term molecular remodeling.

Notably, previous meta-analyses and head-to-head phase II trials have confirmed that mild transient elevation of HGF and IP-10 can also be detected in patients receiving single anti-VEGF monotherapy, indicating partial compensatory activation of these two cytokines represents a universal intraocular feedback response to anti-angiogenic treatment, rather than a molecular marker specific to VEGF/Ang-2 dual blockade (21, 23). The persistent 12-week upregulation observed exclusively in our faricimab-treated cohort may reflect prolonged angiogenic and inflammatory remodeling induced by dual-pathway inhibition, yet direct controlled comparisons with monotherapy groups are still required to validate this specificity.

Correlation analyses revealed a dynamic cytokine network. Before treatment, a tightly interconnected pro-inflammatory/pro-angiogenic network centered around Ang-2, VEGF, HGF, IL-6, and IP-10 existed within nAMD eyes, highlighting the complexity of multifactorial synergy in the pathogenesis of nAMD (21, 24, 25). After faricimab treatment, this network was significantly reconfigured but not completely dismantled; for instance, strong correlations persisted among factors such as HGF, IL-6, and IP-10. Thus, although the primary driving pathways were inhibited, the inflammatory and repair signaling networks within the eye remained active, potentially influencing the long-term disease course and recurrence during treatment intervals.

Consistent with the landmark phase 3 TENAYA and LUCERNE pivotal trials of faricimab, our single-center cohort also achieved prominent anatomical and visual improvements after dual VEGF/Ang-2 inhibition, including significant reductions in CMT and whole retinal thickness accompanied by meaningful BCVA letter gains (26). Those large multicenter registration trials primarily relied on central subfield thickness and standardized BCVA as core efficacy readouts, with limited systematic OCTA quantitative analysis of macular microvasculature, especially superficial foveal capillary density. Our OCTA data further identified a significant decline in superficial foveal vessel density post-treatment, a subtle subclinical vascular alteration rarely characterized in pivotal trial datasets. Critically, large-scale phase 3 studies are constrained by ethical risks, invasive sampling burdens and trial operational limits, making serial aqueous humor collection impossible across thousands of participants. Conventional trial endpoints only reflect macroscopic anatomical and functional improvements without mechanistic molecular evidence to interpret treatment-induced inflammatory-angiogenic remodeling. By profiling 12 intraocular cytokines in paired baseline and 12-week aqueous samples, the current work fills this critical translational gap: we captured sustained compensatory upregulation of HGF and IP-10 following faricimab injection, and validated the direct correlation between elevated IP-10 and foveal capillary rarefaction. Such intraocular molecular signatures cannot be captured by routine OCTA or visual testing in large clinical trials, delivering unique mechanistic and predictive translational value to guide individualized nAMD treatment strategies.

The clinical outcomes of this study were encouraging. OCTA confirmed significant reductions in whole retinal thickness and CMT, which is consistent with previous key clinical trial results (26, 27). Simultaneously, the significant improvement in BCVA established the functional significance of this anatomical benefit. Collectively, these data support faricimab as an effective treatment option for nAM (23).

This study has several limitations. First, it was a single-center investigation with a small cohort (23 nAMD patients and only 10 cataract controls), limiting the stability of baseline cytokine references and the statistical power for subgroup analyses. We failed to stratify patients by macular edema response (complete vs. partial resolution) to compare cytokine differences, nor did we explore whether baseline Ang-2, VEGF and IP-10 could predict anatomical and visual outcomes. Second, aqueous humor was only collected at baseline and week 12 without intermediate sampling at weeks 4 and 8, making it impossible to capture full dynamic cytokine trajectories. Third, no monotherapy anti-VEGF control arm was included, so we cannot distinguish cytokine changes specific to dual VEGF/Ang-2 inhibition from general anti-angiogenic compensatory responses. Larger multicenter longitudinal studies are needed to address these shortcomings. In addition, the 3-month washout threshold defined by our inclusion criteria cannot fully guarantee complete normalization of all intraocular cytokine levels. Though prior literature supports that intravitreal anti-angiogenic agents are mostly eliminated within 12 weeks and most cytokine signals tend to decline after 3 months of drug withdrawal, long-term intraocular inflammatory and angiogenic remodeling triggered by previous anti-VEGF therapy may still persist beyond this interval, potentially introducing minor bias to baseline aqueous cytokine detection. Extended washout periods (at least 6 months) are recommended in future observational biomarker studies to rule out residual treatment-induced cytokine fluctuations and acquire authentic baseline profiles of untreated nAMD patients.

An additional limitation relates to the effect of repeated limbal paracentesis on intraocular inflammatory factors. We only performed two taps separated by 12 weeks. Previous studies show single puncture causes only transient mild ocular irritation with no lasting cytokine changes; the long interval allows temporary inflammatory fluctuations from the first sampling to fully resolve. Frequent serial punctures would induce cumulative anterior segment irritation and alter inflammatory mediator levels, which we avoided by skipping intermediate 4- and 8-week sampling.

Future large-sample multicenter cohorts with sequential aqueous sampling at 4, 8, 12 and 24 weeks are warranted. Subgroup analysis stratified by macular edema remission and predictive models based on baseline Ang-2/VEGF/IP-10 should be performed to identify reliable therapeutic biomarkers. In addition, parallel anti-VEGF monotherapy groups are required to clarify unique molecular signatures of faricimab dual blockade, and multitarget combination therapy targeting HGF or IP-10 may optimize long-term retinal microvascular protection.

## Conclusion

Intravitreal faricimab injection could effectively modulate the intraocular microenvironment in patients with nAMD, rapidly reducing macular edema and improving visual acuity through dual inhibition of the VEGF/Ang-2 pathway. This study also revealed treatment-associated compensatory cytokine responses (HGF elevation) and IP-10 changes potentially related to capillary regulation. These findings not only elucidate the mechanism of action of faricimab but also suggest the potential of aqueous humor cytokine analysis as a novel tool for guiding precision therapy and monitoring treatment responses in nAMD. Future treatment strategies should consider integrating multitarget inhibition to manage this complex disease in a more comprehensive manner.

## Data Availability

The original contributions presented in the study are included in the article/Supplementary material, further inquiries can be directed to the corresponding authors.
