# Supplementary material for: Faricimab in neovascular age-related macular degeneration: aqueous humor cytokine profiling and biomarker correlations
Source: Front Med (Lausanne). 2026 Aug 21;13:1927909. doi: 10.3389/fmed.2026.1927909 (PMC13541480; doi:10.3389/fmed.2026.1927909)
Supplement: Supplementary file 1 [file Table_1.DOCX]

Supplementary Table 1 Correlations between the changed cytokines and OCTA parameters

|  | Ang-2 | FGF basic | HGF | IL-6 | PDGF-AA | TNF-a | IP-10 | HB-EGF | IL-1b | IL-8 | PIGF | VEGF |
| --- | --- | --- | --- | --- | --- | --- | --- | --- | --- | --- | --- | --- |
| SRVD | 0.038(0.865) | -0.248(0.253) | 0.327(0.128) | 0.290(0.179) | -0.016(0.942) | -0.227(0.299) | 0.214(0.326) | 0.406(0.054) | 0.295(0.172) | 0.361(0.090) | 0.179(0.414) | 0.162(0.460) |
| DRVD | 0.124(0.574) | 0.167(0.447) | 0.064(0.773) | 0.350(0.101) | -0.117(0.595) | -0.120(0.586) | 0.181(0.409) | 0.280(0.196) | 0.296(0.170) | 0.335(0.118) | 0.122(0.578) | 0.238(0.274) |
| SFVD | 0.268(0.216) | 0.149(0.497) | 0.320(0.158) | 0.076(0.731) | 0.183(0.403) | 0.247(0.255) | 0.808 (0.000***) | 0.324(0.132) | -0.040(0.855) | 0.143(0.516) | 0.156(0.478) | 0.228(0.294) |
| DFVD | 0.352(0.100) | 0.150(0.493) | 0.107(0.627) | 0.043(0.847) | 0.188(0.390) | -0.214(0.328) | −0.094(0.671) | 0.324(0.131) | 0.241(0.267) | 0.098(0.655) | 0.056(0.799) | −0.308(0.153) |
| FAZ | -0.408(0.054) | -0.305(0.157) | -0.263(0.225) | -0.187(0.392) | -0.210(0.336) | 0.052(0.814) | -0.241(0.268) | -0.225(0.303) | 0.196(0.370) | 0.722(0.000***) | -0.213(0.329) | -0.263(0.226) |
| WRT | 0.521(0.011*) | 0.109(0.622) | 0.271(0.211) | -0.146(0.508) | -0.007(0.973) | 0.154(0.484) | 0.040(0.857) | 0.014(0.951) | -0.266(0.219) | -0.063(0.776) | 0.321(0.136) | 0.443( 0.034*) |
| CMT | 0.729((0.000***) | 0.207(0.344) | 0.272(0.209) | 0.077(0.727) | -0.084(0.703) | 0.327(0.128) | 0.202(0.355) | 0.219(0.314) | -0.056(0.801) | 0.051(0.816) | 0.403(0.057) | 0.523( 0.010*) |

Values in each cell represent Spearman’s correlation coefficient (r), with corresponding P-values shown in parentheses. BCVA, best-corrected visual acuity; SRVD, superficial retinal vessel density; DRVD, deep retinal vessel density; SFVD, Superficial foveal vessel density; DFVD, deep foveal vessel density; WRT, Whole retinal thickness; CMT, central macular thickness. *indicates p<0.05, ** indicates p<0.01, *** indicates p<0.001.
